# Viroporins Manipulate Cellular Powerhouses and Modulate Innate Immunity

**DOI:** 10.3390/v16030345

**Published:** 2024-02-23

**Authors:** Leticia Cedillo-Barrón, Julio García-Cordero, Giovani Visoso-Carvajal, Moisés León-Juárez

**Affiliations:** 1 Department of Molecular Biomedicine, Center for Research and Advanced Studies (CINVESTAV-IPN) Av., IPN # 2508 Col., San Pedro Zacatenco, Mexico City 07360, Mexico; jugarcia@cinvestav.mx (J.G.-C.); giovani.visoso@cinvestav.mx (G.V.-C.); 2 Escuela Superior de Medicina, Instituto Politécnico Nacional, Salvador Díaz Mirón esq, Plan de San Luis S/N, Miguel Hidalgo, Casco de Santo Tomas, Mexico City 11340, Mexico; 3 Instituto Nacional de Perinatología Isidro Espinosa de los Reyes, Mexico City 11000, Mexico; moisesleoninper@gmail.com

**Keywords:** viroporins, mitochondria, innate immune response, inflammasome

## Abstract

Viruses have a wide repertoire of molecular strategies that focus on their replication or the facilitation of different stages of the viral cycle. One of these strategies is mediated by the activity of viroporins, which are multifunctional viral proteins that, upon oligomerization, exhibit ion channel properties with mild ion selectivity. Viroporins facilitate multiple processes, such as the regulation of immune response and inflammasome activation through the induction of pore formation in various cell organelle membranes to facilitate the escape of ions and the alteration of intracellular homeostasis. Viroporins target diverse membranes (such as the cellular membrane), endoplasmic reticulum, and mitochondria. Cumulative data regarding the importance of mitochondria function in multiple processes, such as cellular metabolism, energy production, calcium homeostasis, apoptosis, and mitophagy, have been reported. The direct or indirect interaction of viroporins with mitochondria and how this interaction affects the functioning of mitochondrial cells in the innate immunity of host cells against viruses remains unclear. A better understanding of the viroporin–mitochondria interactions will provide insights into their role in affecting host immune signaling through the mitochondria. Thus, in this review, we mainly focus on descriptions of viroporins and studies that have provided insights into the role of viroporins in hijacked mitochondria.

## 1. Introduction

Viruses are obligate intracellular parasites and possess a smaller genome than mammalian cells; thus, viral genomes encode a small number of proteins. Viruses must optimize the function of their proteins to successfully complete the viral replication cycle. Both structural and nonstructural viral proteins perform many functions during the viral cycle to evade all mechanisms that restrict viral replication. Among these proteins, viroporins are molecules present in a wide range of viruses which modulate and manipulate the cell environment by disrupting the balance between the intracellular ions (e.g., Na^+^, K^+^, Ca^++^, Cl^−^, and H^+^) through the formation of pores in various cell organelles [1].

Viroporins are a family of small (usually up to 120 amino acid residues), hydrophobic proteins that have attracted tremendous interest since they were first described. They typically contain one or more hydrophobic sequences and have self-oligomerization abilities to form transmembrane central hydrophilic channels that can transport small ions [2,3]. Viroporins are essential molecules in the viral cycle and are crucial for pathogenesis. A single viroporin may simultaneously participate in different steps of the viral cycle, such as entry, uncoating, replication, assembly, and release, thus ensuring an ideal microenvironment [4,5]. Furthermore, a single virus can encode more than one viroporin. Simian virus 40 (SV40) encodes three viroporins, VP2, VP3, and VP4 [4]; DENV encodes two viroporins, NS2A and NS2B [6,7]; and the recently characterized SARS-CoV-2 encodes the E and ORF3a viroporins [8].

Viroporins have been identified in a wide range of both RNA and DNA viruses. However, the largest number of viroporins has been described in RNA viruses, and they are involved in the modulation and manipulation of the cell environment through the induction of multiple cellular phenomena, such as immune response, inflammasome activation, membrane remodeling, autophagy induction, alteration of mitochondrial functions, vesicular trafficking, and apoptosis [4,7,9,10,11].

**Figure 1:**
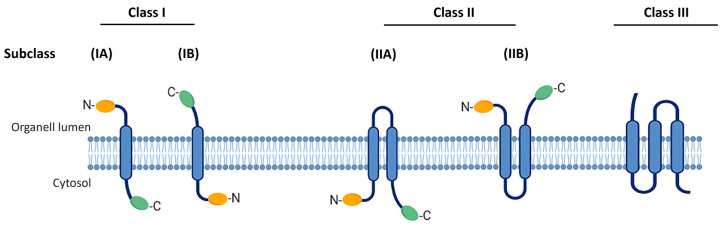
Classification of viroporins. Based on the number of TMDs, there are three types of viroporins (I, II, and III). Their subclassification is based on transmembrane topology of extreme N- and C-terminal ends either in the lumen of the cell organelles or in the cytosol (A and B). TMD, transmembrane domain.

The classification of viroporins is currently based on the number of transmembrane domains (TMDs) as described in Figure 1 while its subclassification is based on transmembrane topology, depending on whether the N- and C-terminal ends are in the lumen of the cell organelles or in the cytosol. The current classification system includes three classes of viroporins (I, II, and III) and their respective subclasses. Type I viroporins possess only one TMD and are further subclassified into 1A and 1B. Viroporins from subclass 1A have an N-terminal domain of 9–25 amino acids exposed towards the lumen of the cell organelles and a cytosolic tail (50 amino acids) at the C-terminus, which is prone to phosphorylation [1]. Viroporins from subclass 1B are characterized by an N-terminal domain located in the cytosol and a terminal tail at the C-terminus that is exposed to the cell organelle lumen. Class II viroporins contain two TMDs linked by a loop of basic amino acids and are subclassified into IIA and IIB based on topology [3]. Class III viroporins contain three TMDs; however, to date, no corresponding subclasses have been established as shown in Figure 1 [1,12].

**Figure 2:**
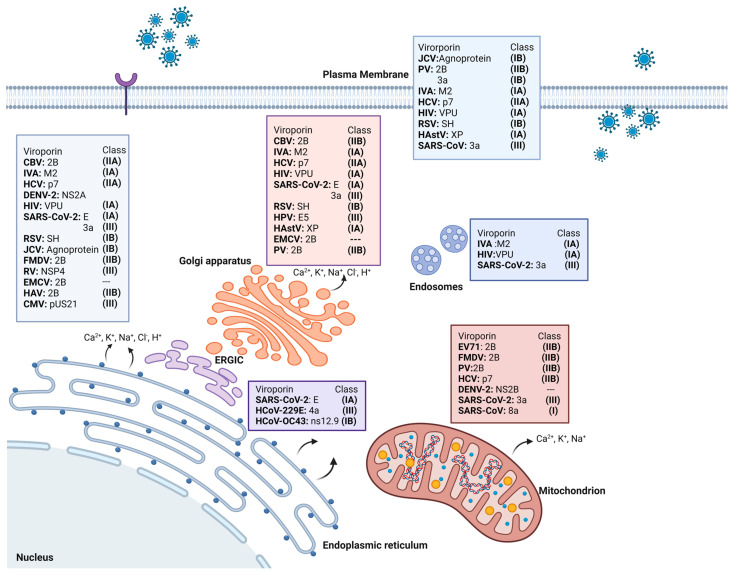
Different viruses with their respective viroporins, their classification, and localization in the different organelles where they are anchored. CBV: Coxsackie B3 virus. PV: poliovirus. JCV: John Cunningham virus. EV71: enterovirus 71. FMDV: foot-and-mouth disease virus. HastV: human astroviruses.

Mitochondria are an important target for many viral proteins, including viroporins, which in turn may have an impact on the membrane potential and mitochondrial stress, owing to the imbalance in reactive oxygen species (ROS), leading to repercussions in antiviral immunity [13]. The viroporins promote the selective release of various ions based on the membranes targeted, affecting the cell in diverse ways. If viroporins are inserted into mitochondrial membranes, the function of the mitochondria as signaling centers for innate immunity may be compromised. The effect of viroporins on mitochondria is not always direct, but there is an intense crosstalk between mitochondria and different mediators, which indicates the importance of the role of mitochondria [13,14]. Figure 2 shows several examples of different viroporins. The channels resulting from viroporins can be functionally selective, nonselective, voltage dependent, or voltage independent.

Among other factors, intracellular homeostasis depends on the ability to regulate the exchange of ions and solutes between the cell membranes. Various ions are necessary to generate the electrochemical gradients facilitating different cellular functions, such as the regulation of gene expression and cell pH [8,15]. In this review, we summarize the characteristics of viroporins and their roles during the viral cycle and describe the importance of mitochondria as key organelles in cellular functions. Finally, we focus on viroporins that target the mitochondria and their impact on innate immunity.

## 2. Role of Viroporins in the Viral Cycle

Viroporins are essential for viral replication and pathogenesis. Furthermore, considerable evidence exists regarding the distinct roles of viroporins in the viral cycle. However, it is difficult to define their exclusive roles in the entry, replication, or exit of the virus because of the multifunctionality of the viral proteins [1,16,17]. The first step in order for most viruses to enter host cells is the interaction between the viral proteins and host surface molecules [18]. Thus, some viruses utilize viroporins to permeabilize the host cell membrane [19]. The influenza virus A (IVA) is the causative agent of one of the most serious respiratory illnesses. The genome of this virus encodes viroporin, M2, which is one of the most emblematic and widely characterized viroporins involved in viral entry [20]. This protein functions as a proton-conducting channel in the viral envelope that induces acidification of the virus interior within endosomes, promoting a conformational change in the envelope glycoprotein hemagglutinin [21]. This, in turn, induces fusion of the viral envelope with the endosomal membrane, encouraging the delivery of the nucleoprotein complex into the cytoplasm [22,23]. Another interesting example is the p7 viroporin from hepatitis C virus (HCV), which prevents acidification in the otherwise acidic intracellular compartments of the infected cells [24].

Each virus establishes its budding area; some use the plasma membrane (IVA and HIV-1), while others use cytosolic organelle membranes, such as the endoplasmic reticulum (ER)–Golgi intermediate compartment (ERGIC) for SARS-CoV-2 and ER for HCV. Additionally, the inside of the infected host cell may act as the budding area, as described for Vpu, an HIV viroporin that is expressed late in the viral life cycle and possesses a single transmembrane domain with channel properties that improve viral particle release [25].

Human cytomegalovirus (CMV) decreases intracellular calcium stores via pUS21, a protein expressed during the late stages of the viral cycle. It serves as an ion channel to reduce intracellular ER storage of Ca^2+^ and protects cells against apoptosis through a BAX-inhibitory domain that interferes with the induction of apoptosis in infected cells [26]. Similarly, the 6K protein of the Sindbis virus decreases Ca^2+^ levels in the ER and Golgi apparatus. The individual expression of the PV2B protein inhibits protein trafficking via the Golgi apparatus. Other organelles are also affected, such as the cytoplasmic membrane, where depolarization due to an altered electrochemical gradient facilitates the release of new viral particles. Many viruses exploit this ability to induce ion release, thereby assisting the viral cycle. This was also observed for SARS-CoV-2 which encodes two viroporins, E and ORF3a [9,27,28]. Both are localized on the ER and Golgi apparatus, where they increase the permeability to Ca^2+^ and promote alkalinization to facilitate replication and the corresponding viral progeny set [29]. Figure 2 shows many examples of viroporins that target different membranes.

## 3. Importance and Functions of the Mitochondria

Mitochondria are double-membrane cell organelles with an outer and inner membrane. The outer membrane is responsible for ion exchange and transport of small molecules through proteins, such as voltage-dependent anionic channel proteins, and translocases, such as the TOM/TIM complex. In contrast, the inner membrane possesses selective permeability and is a site for oxidative phosphorylation (OXPHOS). Mitochondria play crucial roles in the cell, including energy production, regulation of calcium signaling, regulation of ROS, and maintenance of cellular homeostasis, by coordinating several functions via cellular and molecular mechanisms [30,31,32]. Furthermore, mitochondria play an important role in innate immunity during viral infections; they also regulate the activation of the inflammasome, which culminates in pyroptosis and apoptosis [33]. Therefore, many viral proteins target the mitochondria, inducing changes and dysfunction in mitochondrial membrane potential, inducing mitochondrial stress, and increasing ROS levels [13].

The strategy of many viruses is to target and interfere with mitochondrial homeostasis through molecules involved in innate immunity, such as NLRX1, TRAF6, NLRP3, and IRGM mitochondrial antiviral signaling protein (MAVS). This is an efficient strategy because it not only targets the mitochondria, but also cellular defense mechanisms [34,35,36]. This is necessary, as the detection of viral infection in cells triggers metabolic changes from mitochondrial OXPHOS to glycolysis to fight the pathogen [37]. OXPHOS is activated during the establishment of an interferon (IFN)-mediated antiviral state. Likewise, the participation of certain mitochondrial enzymes during viral infection has been described, such as methyl crotonyl-CoA carboxylase, which is associated with TRAF6 and results in greater MAVS-dependent signaling, inducing a large antiviral microenvironment [38]. This has facilitated an understanding of how viruses manipulate the mitochondrial metabolism. However, the cell responds to fight infection by activating certain metabolic genes, which also leads to inflammation, in an attempt to contain viral infection. This cellular process involves the canonical function of the mitochondria to promote ATP production.

## 4. Mitochondria and Their Immunoregulatory Role in Viral Infections

Mitochondria play an important role in establishing an antiviral immune response and are important targets for structural and nonstructural viral proteins. Viral proteins directly or indirectly disturb the mitochondria and induce changes in the mitochondrial dynamics and function in host cells. Thus, they successfully contribute to viral propagation via mitochondrial dysfunction [33,39,40].

Notably, viruses have developed many strategies to utilize mitochondria for their benefit and successful viral replication; for instance, the herpes simplex virus depletes host mitochondrial DNA [41]. Some viral proteins, such as viroporins, promote the selective release of several ions, affecting the cell differently, depending on the altered cell components.

All viruses contain different pathogen-associated molecular patterns (PAMPs) that are sensed by cellular molecules called pattern recognition receptors (PRRs) [42]. After the virus is recognized, a complex intracellular signaling cascade occurs, and transcription factors, such as regulatory factors, interferon regulatory factor 3 (IRF3) and IRF7, are activated and induce the production of type I and III IFN, which in turn induce more than 700 antiviral molecules that together form the interferome, as well as the production of proinflammatory cytokines [43]. To induce an antiviral microenvironment to inhibit viral replication and prevent the spread of infection to neighboring cells [44], the innate immune response uses three primary PRRs to detect viruses: retinoic acid-inducible gene-1 (RIG)-1 like receptors (RLRs), Toll-like receptors (TLRs), and NOD-like receptors (NLRs) [45].

Mitochondria react to viral infections by releasing damage-associated molecular patterns (DAMPs), such as n-formyl peptides, cardiolipin, ROS, and unmethylated mitochondrial DNA (mtDNA), which are recognized by innate immune system receptors and trigger an immune response. Mitochondria can initiate an innate immune response via three different signaling pathways: the RIG-1/MAVS, NLRP3, and TLR9 [33]. RLR helicases can detect double-stranded RNA segments. Some examples of RLR helicases include RIG-1, melanoma differentiation-associated gene-5 (MDA5), and laboratory of genetics and physiology 2 (LGP2) [46]. Helicases comprise a C-terminal domain that binds to viral RNA, and an N-terminal domain that contains two caspase activation and recruitment domains (CARDs) [47]. They also possess ATPase activity, which allows them to traverse through double-stranded RNA to expose hidden CARDs [48]. This, in turn, allows the E3 ubiquitin ligases TRIM25 and RIPLET to recognize the RNA 5ʹ-triphosphate structure, releasing CARD from the regulatory domain. This conformational change allows the two CARD domains of RIG-1 or MDA5 to interact with the CARD of MAVS, which is also known as the RIG-1 signaling adaptor protein [49]. MAVS acts as a sentinel on the external mitochondrial membrane and a signaling link by detecting viral RNA upstream and downstream signaling via IRF3 and NF-κB to facilitate the expression of multiple proteins involved in the antiviral response, inflammatory response, autophagy, and cell death. MAVS also activates the stimulator of interferon genes (STING) and mediates the activation of two non-canonical IκB kinases (IKKs), TANK-binding kinase 1 (TBK1), which phosphorylates IRF, and inducible IKK, which binds to interferon-sensitive response elements (ISREs) in the nucleus [50,51,52,53,54,55].

The DAMPs, released into the cytosol during viral mitochondrial aggression, are mtDNA, which is sensed by cGAS, triggering the cGAS-STING pathway and activating the STING-IRF3 pathway, which further culminates in a type I IFN response. However, mtDNA is similar to bacterial DNA: both molecules share hypomethylated motifs that can be detected by TLR9, which recognizes CpG sequences in endosomes and triggers a signaling cascade via MYD88 or TRIF [56,57].

**Figure 3:**
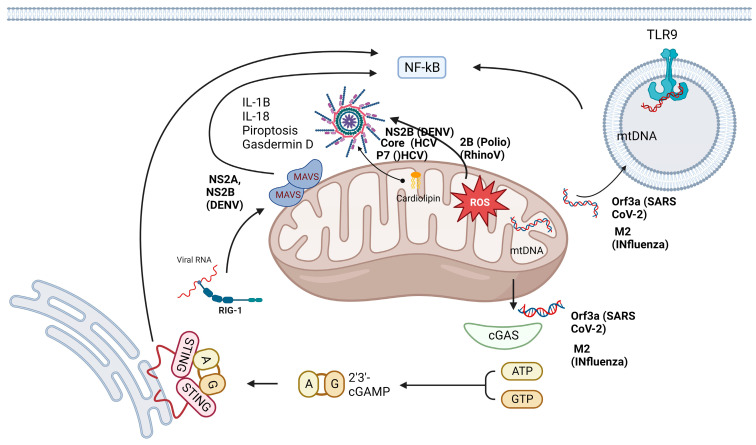
Mitochondria are hubs for antiviral signaling and are targets for viroporins. The mitochondria powerhouse plays a key role in the innate immune response, as it participates in the main pathways involved in the immune response: the TLR, RLR and NLR pathways. However, several viroporins, such as P7, NS2A, NS2B, core, M2, and Orf3a, can directly or indirectly challenge mitochondria. Some of them can permeabilize the mitochondrial membrane and allow the release of DAMPs, such as cardiolipin, mtDNA and ROS, which in turn activate different molecules, such as TLR9, cGAS, and inflammasomes. All these signaling pathways induce the production of inflammatory cytokines that culminate in the pathogenesis of viral infections. mtDNA, mitochondrial DNA; NLR, NOD-like receptor; RLR, RIG-1-like receptor; ROS, reactive oxygen species; TLR, Toll-like receptor.

Mitochondrial ROS (mtROS) are DAMPs released during viral insults that activate the NLRP3 inflammasome [58]. Previous studies have highlighted that one of the primary functions of mtROS is the amplification of MAVS-induced signaling. The regulation of mtROS has been described previously: the metabolic sensor 5′-AMP-activated protein kinase (AMPK) inhibits the generation of mtROS, and the hypoxia-inducible factor (HIF-1α) regulates the expression of mtROS [59,60], as shown in Figure 3.

## 5. Interference of Viroporins with Mitochondrial PRRs

According to the above-mentioned information, viroporins are proteins encoded by the viral genome that alter cell membrane permeability and trigger subsequent signaling. They hijack host cell defenses by altering mitochondrial dynamics, disrupting membrane potential, promoting the release of ROS, inducing hypoxia, and altering intracellular calcium levels [61].

As mentioned above, viroporin M2 of IVA induces the translocation of mtDNA to the cytosol in a MAVS-dependent manner, whereas the viral NS1 protein binds to this mtDNA to evade the STING-mediated antiviral immune response because mtDNA activates cGAS to trigger this response [62]. Viroporins act at different levels in the mitochondria, either in proviral or antiviral processes, as described in Table 1, where it is shown how it affects the mitochondria.

Mitofusin 2 (MNF) is in the outer mitochondrial membrane; it is considered a dynamin-like GTPase and serves to regulate mitochondrial fusion and cell metabolism [77]. MFN2 is another important target of viroporins in the regulation of the MAVS signaling pathway as it interacts directly with MAVS and competitively prevents the interaction of RIG or MDA5 with MAVS, thereby reducing antiviral immunity [78]. Certain viroporins also act as a complex, such as NS2B viroporin and NS3 viral protease of DENV, which cleave mitofusin 1 and 2 and affect mitochondrial fusion [73].

In contrast, Chatel et al., 2016, showed that DENV can block the antiviral signaling induced by mitochondria through the NS4B protein (which may be a viroporin), inducing mitochondrial elongation. After inactivating the fission factor DRP1, mitochondria elongation alters the mitochondria-associated membranes, resulting in damage to the interferon response promoting viral replication [79].

Viroporins can also manipulate mitophagy by phosphorylating Drp1 and overregulating Parkin and PINK1, leading to persistent infection [80]. Another strategy used by viruses to establish a persistent infection is to control the mitochondrial metabolism by manipulating the glycolytic pathway or the TCA cycle [81].

## 6. Mitochondrial ROS and Viroporins

Oxidative stress is the result of excess ROS (i.e., an imbalance between ROS accumulation and the antioxidant defense systems) [82]. However, basal ROS levels are mediators that activate cell signaling pathways, and excessive ROS production results in cell and tissue damage because these molecules can react with proteins, lipids, and nucleic acids [82,83]. The primary cellular source of ROS is the mitochondria; during intense oxidative metabolism, mitochondria generate and sequester ROS. Furthermore, 2% of molecular oxygen uptake by cells during respiration is converted into ROS (mtROS). During the respiratory chain, the flow of electrons through mitochondrial complexes I, II, and III promotes the generation of these molecules [84]. In a hypoxic environment, mtROS, such as H_2_O_2_, can stimulate pathways involving c-Jun kinase, p53, and NF-κB, resulting in the stimulation of genes regulating hypoxia homeostasis [85].

The mechanisms by which mtROS mediate the regulation of signal transduction pathways have been characterized. ROS, at elevated levels, can interact with specific regions of proteins by oxidizing the cysteine and methionine motifs [86]. The Kelch-like ECH-associated protein 1 (Keap1)/nuclear factor E2-related factor 2 (Nrf2) signaling system is among the primary regulatory pathways activated by ROS via the mechanisms. The destruction of Keap1 by ROS leads to the dissociation of the Nrf2–Keap1 complex and the activation of Nrf2 [87].

A connection between mtROS and innate immunity has also been established. The mechanism by which mtROS regulates the activation or signaling of TLR, RLR, and NLR, and the production of cytokines, such as TNF-α, reveals that mtROS can mediate an excellent immediate immune response against pathogens [88].

Viruses have demonstrated several strategies for the use of mitochondria as they are important sources of mtROS owing to their fundamental role in respiration. However, mtROS have been described as a cellular biological weapon with innate effector functions during infection, affecting the multiplication and production of viral progeny. Thus, ROS are produced during the response to infection, stress, damage, or by certain viroporins [89]. Thus, the generation of mtROS must be controlled because, although they induce an efficient immune response at low concentrations, they cause mitochondrial damage and sustained inflammation at high concentrations, leading to pathological consequence, Figure 3 shows examples of the impact of viroporins on mitochondria.

Tian et al., 2021, found that the Orf3a viroporin of SARS-CoV-2 induces mitochondrial damage, releasing mtROS to promote HIF-1α expression and facilitate infection and proinflammatory cytokine production as shown Figure 3 [90]. Furthermore, Ma et al. demonstrated that the IVA M2 viroporin exhibits proton channel activity and induces ROS production required to antagonize autophagy and amplify the MAVS signaling pathway [91]. Lee et al., 2020, described that HCV has two viroporins: P7, which permeabilizes the mitochondrial membrane via interaction with phosphatidyl serine (a negatively charged phospholipid) located in the lipid rafts of membranes, and another HCV core viroporin, which is located in the outer mitochondrial membrane and decreases with an increase in ROS production [71,92].

One clear example of how viruses can induce a productive or non-productive infection by modulating mtROS generation was described by Ojeda et al. (2018), while studying HIV infections in astrocytes. During productive infection, the virus can attenuate the generation of mtROS, whereas in a non-productive state, the virus induces high mtROS production, causing mitochondrial damage and inducing cell death via inflammasome activation [93].

Finally, the Parkin protein (a key player in mitophagy) regulates the activation of the mtROS-mediated NLRP3 inflammasome during viral infections, as it inhibits the antiviral response. In conclusion, mtROS play a critical role in regulating the autophagy–inflammasome axis during the innate immune response to viral infections [94].

## 7. Role of Viroporins in Activating the Inflammasome and Mitochondria

The inflammasome is a molecular platform comprising a multiprotein complex that regulates the maturation of proinflammatory cytokines, such as the pro-forms of IL-1β and IL-18. These pro-forms are synthesized via the initial stimulation of the TLR or RLR pathways by PAMPs or DAMPs (first signal) and are activated and secreted through posttranslational processes triggered by the activation of the inflammasome (second signal), which in turn activates the proteolytic function of caspase 1 [95,96]. The inflammasome regulates at least three protective responses within the host cell: the secretion of proinflammatory cytokines (IL-1β and IL-18), the formation of pores by gasdermin D, and the induction of a type of cell death termed “pyroptosis” [97,98] NLR inflammasomes are structurally formed by the N-terminal effector, the central NACHT, and the C-terminal leucine-rich repeat domain. The effector domains can be acidic transactivation, baculovirus inhibitory repeats, CARD, pyrin domain, or a non-homologous domain [99].

Inflammasomes are categorized into four classes based on their effector domains: NLRP1/NALP1b, NLRC4/IPAF, NLRP3/NALP3, and AIM-2 [100,101]. However, those that are primarily responsible for activating antiviral responses include AIM-2 and NLRP3. They can be activated by a complete virus or part of a virus containing PAMPs or DAMPs [100]. They can also be activated by viral proteins that act as viroporins [102].

The disruption of Ca^2+^ and K^+^ levels by viroporin ion channels acts as a secondary signal that induces inflammasome activation. Three activation pathways for the NLRP3 inflammasome by viroporins have been described (M2 of IVA): the downregulation of Ca^2+^ homeostasis (2B of polio- and rhinoviruses), ROS production (3a of coronavirus) [10], and H^+^ homeostasis modification via ion channel activity by the best-characterized viroporin the M2 protein of IVA, which activates the inflammasome by altering the concentration of ions (including ROS) in intracellular compartments [62].

The 2B protein of rhinovirus can induce the formation of pores in the membranes of the ER and Golgi apparatus, thus decreasing the levels of Ca^2+^ and H^+^ in these organelles [103]. Additionally, the viroporin SH of human respiratory syncytial virus (RSV) accumulates inside the trans-Golgi network in the lipid rafts, where it forms Na^+^ and K^+^ channels. Both viroporins activate inflammasomes by downregulating ion production [104].

The P7 protein of CSFV can form pores in the membrane and downregulate Ca^2+^ homeostasis, thereby activating the NLRP3 inflammasome [105]. Viroporin 2B from the encephalomyocarditis virus increases the concentration of Ca^2+^ in the cytoplasm, promoting the secretion of IL-1β [11].

Viroporins, such as the Vpu protein of HIV, not only disrupt membrane permeability by deregulating the homeostasis of different ions but can also form selective voltage-gated proton channels [106,107]. The HCV P7 protein destabilizes the membranes of intracellular vesicles in a pH-gated proton-channel manner [92]. Two proteins of SARS-CoV-2, envelope protein and Orf3a, reportedly exhibit viroporin activity; these proteins can create ionic channels [108]. Notably, RNA viruses are not the only viruses that can activate the inflammasome; some DNA viruses, such as vaccinia virus and mouse CMV, can also activate the AIM-2 inflammasome in a caspase-1-dependent manner as is shown in Figure 3 [109].

Overall, the deletion or silencing of viroporin-related genes can strongly affect viral progeny, which is reflected in the viral pathogenicity. Therefore, these genes are strong candidates for vaccines and as therapeutic targets [1,17].

## 8. Viroporins as Targets for Viral Treatment

As we have mentioned above, the viroporins have a key role during the different steps of the viral cycle, such as viral entry into target cells, replication, assembly and maturation, and viral budding. Another important role of the viroporins is their involvement in viral immunopathogenesis [110,111]. Many important pathogenic viruses, such as Ebola, SARS-CoV, MERS, SARS-CoV-2 (pandemic virus), influenza A, human immunodeficiency virus 1, and HCV, comprise at least one viroporin. A clear example of the role of viroporins during the viral cycle was observed when Mandala et al. deleted the E protein of SARS-CoV and SARS-CoV-2, which provoked a reduction in viral titer by inhibiting virus maturation [112,113].

Clearly, being the viroporins essential for a myriad of events, blocking the activity of viroporins will have an impact on the viral cycle and possibly immunopathogenesis. Therefore, viroporins are positioned as exceptional targets for the treatment of viral diseases [112,114]. Strategies such as mutation of the viroporin activity or deletion of this molecule are suitable. However, the use of anti-viroporin drugs is the more commonly implemented strategy.

Amantadine (AM2) was the first drug to be used to inhibit influenza virus replication through the inhibition of viroporin activity in the ion channel of the M2 molecule, preventing proton conduction and inhibiting viral entry [115]. Its efficacy was evaluated for other viruses, such as HCV p7, FMDV 2B, SARS-CoV [116]. It was also used during the SARS-CoV-2 pandemic. However, drug resistance limited its use. Thus, other ion channel inhibitors (pharmacophores), among the aromatic heterocyclic compounds that can strongly bind to proteins, were investigated, such as the spirene guanidine analogue, 2-[3-azaspiro (5,5) undecanol]-2-imidazoline (BL-1743) [117]. Other molecules, such as tauroursodeoxycholic acid as hexamethylene amiloride [118], gliclazide [119], memantine and other flavonoids such as epigallocatechin and quercetin were also effective [112].

In summary, viroporins represent a suitable alternative for the design of antiviral drugs, using novel research tools to dissect the viroporin function.

## 9. Discussion

Viral diseases present a substantial challenge to human health. Several viroporins reportedly function as important elements of viral pathogenesis. These viral molecules represent a biological advantage for viruses and a critical strategy to facilitate host cell manipulation. Viroporins change cellular permeability by forming hydrophilic pores in different host cellular membranes and disturbing the balance of the intracellular ions (e.g., Na^+^, K^+^, Ca^++^, Cl^−^, and H^+^). The resulting imbalance often causes the activation of innate immune responses, where mitochondria play an important role. The mitochondria are a hub component in the regulation of several phenomena of innate immunity and in orchestrating the immune response against any insult, such as those caused by a viral infection, as well as viral proteins, particularly those that possess viroporin activity. Viroporins target host cell mitochondrial cells to increase ATP production as part of a strategy to benefit the virus; however, mitochondria also release several DAMPs that not only confer an overall protective advantage but also initiate the pathogenic response. One recent report shows that the mitochondria in SARS-CoV-2 encode three viroporins that target cell membranes, including the mitochondrial membrane. Induced mROS production activates the release of mitochondrial DNA via the NIM811-sensitive mitochondrial-permeability pore, thereby activating inflammasome and cytokine secretions [75]. The mature M protein of the DENV is a known viroporin that functions as an ion channel involved in virus entry M.A. [120]. Additionally, Shrivastava et al. suggested that another viroporin-like protein of DENV, NS2A, may be involved in intracellular Ca^2+^ homeostasis or mitochondrial disruption, thereby activating the NLRP3 inflammasome and inducing IL-1β overproduction [7]. Although considerable information is available regarding the role of viroporins during the viral cycle, recent studies have focused on mitochondria as an organelle that is directly and indirectly affected by viroporins. Given the uncertainties of viroporins, the detailed future scope of studies can be used to fill the gaps in the literature. Nevertheless, the available scientific evidence suggests that it is imperative to develop new anti-viroporin drugs to effectively combat viral infections.

## 10. Conclusions

- Viroporins target the mitochondria and alter membrane potential, as well as the concentration of reactive oxygen species and, importantly, calcium homeostasis.
- Viroporins are molecules involved in the cellular viral cycle and are strongly involved in pathogenesis.
- A direct link between mitochondria and innate immune signaling has been described.
- Consequently, the interaction between viroporins and mitochondria will interfere in the process of innate immunity.

## Figures and Tables

**Table 1:** Viroporins and their impact on the mitochondria.

Virus	Viroporin	Class and Subclass	Function	References
Coxsackievirus (CBV)	2B	IIA	2B reduces [Ca^2+^]ER and [Ca^2+^] Golgi in HeLa cells, this leads to a reduction in the mitochondrial Ca2+ uptake. Also confer an antiapoptotic state to infected cells to suppress infection-limiting apoptotic host cell responses.	[63,64,65]
Poliovirus (PV)	2B	IIB	2B Induces reorganization of the mitochondrial network and induces an anormal perinuclear distribution. Cytochrome *c* release, suggesting involvement of the mitochondrial pathway in viroporin-induced apoptosis.	[65,66,67]
Foot-and-mouth disease virus (FMDV)	2B	IIB	Induction of mtDNA release through a mechanism involving Mitochondrial permeability transition pore (mPTP) and activation cGAS-mediated antiviral response.	[65,68]
Enterovirus 71 (EV-71)	2B	---	Mitochondrial localization of the 2B protein (by the C-terminal region, amino acids 63–80) induces apoptosis by interacting directly with and recruiting the proapoptotic protein Bax and inducing Bax conformational activation.	[65,69,70]
Hepatitis C Virus (HCV)	p7	IIB	p7 induce mitochondrial depolarization and ATP depletion. Affect apoptosis pathways through alteration of permeability in the mitochondrial membrane.	[71,72]
Dengue virus (DENV)	NS2A NS2B	--- ---	Induce changes in mitochondrial morphology, presenting fragmented mitochondria, elongated shapes with perinuclear localization, in addition to a decrease in membrane potential (ΔΨm). Role in NLRP3 inflammasome activation. NS2B-NS3 cleave mitofusin 1 and 2 and affect mitochondrial fusion.	[7,73]
SARS-CoV-2	E 3a	I III	E and 3a protein increase Ca++ flux into the cytosol where it is taken up by the mitochondrion, then, mROS are produced, which oxidizes the mtDNA, which is released through the mtPTP to bind to the NLRP3 inflammasome. 3a induces robust mitochondrial fragmentation.	[74,75,76]
SARS-CoV	3a 8a	--- ---	3a protein disrupts intracellular ionic concentrations and causes mitochondrial damages, thereby activating the NLRP3 inflammasome. 8a protein is located in the mitochondria, where it can perturb mitochondrial membrane potential and induce apoptosis through a caspase-3-dependent pathway.	[9,10]
Influenza Virus A (IVA)	M2	IA	IVA induces the translocation of mtDNA to the cytosol in a MAVS-dependent manner.	[62]

## Data Availability

Not applicable.
